# Color-preserving passive radiative cooling for an actively temperature-regulated enclosure

**DOI:** 10.1038/s41377-022-00810-y

**Published:** 2022-05-04

**Authors:** Yining Zhu, Hao Luo, Chenying Yang, Bing Qin, Pintu Ghosh, Sandeep Kaur, Weidong Shen, Min Qiu, Pavel Belov, Qiang Li

**Affiliations:** 1 State Key Laboratory of Modern Optical Instrumentation, College of Optical Science and Engineering, Zhejiang University, 310027 Hangzhou, China; 2 Key Laboratory of 3D Micro/Nano Fabrication and Characterization of Zhejiang Province, School of Engineering, Westlake University, 18 Shilongshan Road, 310024 Hangzhou, Zhejiang Province China; 3 Institute of Advanced Technology, Westlake Institute for Advanced Study, 18 Shilongshan Road, 310024 Hangzhou, Zhejiang Province China; 4 Department of Physics and Engineering, ITMO University, Saint Petersburg, Russia

**Keywords:** Optical materials and structures, Applied optics

## Abstract

Active temperature control devices are widely used for the thermal management of enclosures, including vehicles and buildings. Passive radiative cooling has been extensively studied; however, its integration with existing actively temperature regulated and decorative enclosures has slipped out of the research at status quo. Here, we present a photonic-engineered dual-side thermal management strategy for reducing the active power consumption of the existing temperature-regulated enclosure without sacrificing its aesthetics. By coating the exterior and interior of the enclosure roof with two visible-transparent films with distinctive wavelength-selectivity, simultaneous control over the energy exchange among the enclosure with the hot sun, the cold outer space, the atmosphere, and the active cooler can be implemented. A power-saving of up to 63% for active coolers of the enclosure is experimentally demonstrated by measuring the heat flux compared to the ordinary enclosure when the set temperature is around 26°C. This photonic-engineered dual-side thermal management strategy offers facile integration with the existing enclosures and represents a new paradigm toward carbon neutrality.

## Introduction

**Fig. 1:**
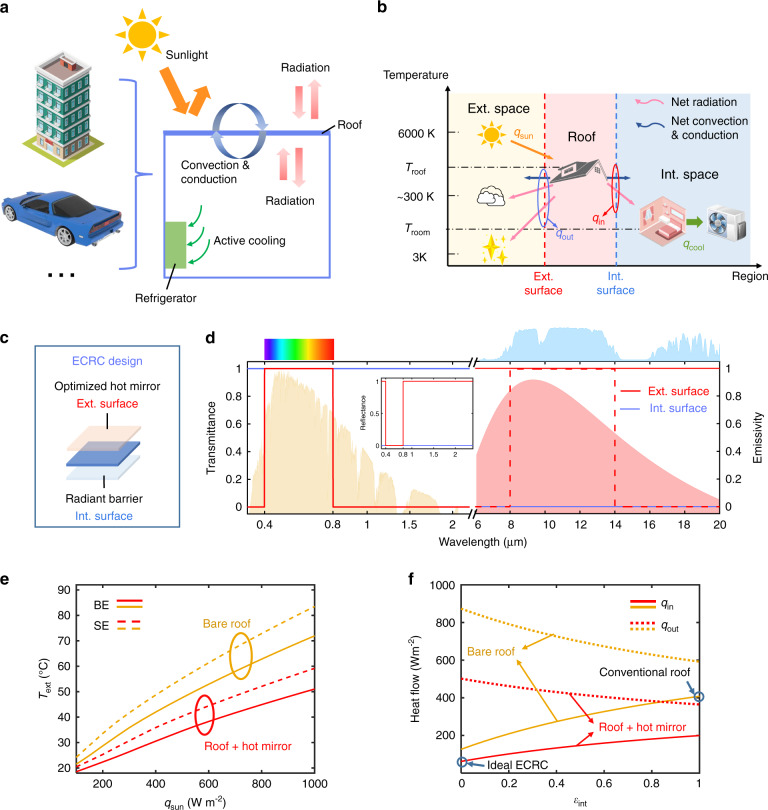
The working principle of the enhanced color-preserving radiative cooling (ECRC) strategy for thermal management of actively temperature-regulated enclosures. **a** Schematic diagram of the heat exchange between an actively temperature-regulated enclosure and external space via the roof. **b** Energy flow diagram of the overall system at thermal steady-state. **c** Design of the ECRC. The structure consists of two parts: a visible-transparent optimized hot mirror on the exterior surface of the roof and a visible-transparent radiant barrier on the interior surface. **d** Ideal spectra for the exterior surface (red) and the interior surface (blue) of the ECRC. The red dashed and solid lines denote the selective emitter (SE) and the broadband emitter (BE), respectively, for the exterior surface. The visible band ranges from 0.4 to 0.8 μm. The orange area denotes AM 1.5 solar spectrum. The pink area denotes the radiation spectrum of a blackbody at 35 °C. The light blue area denotes the atmosphere window. **e** Calculated exterior surface temperature (*T*_ext_) of roofs illuminated by different solar irradiance (*q*_sun_). The interior surface emissivity is set as 0.9. **f** Calculated outward (dotted line) and inward (solid line) heat flow of roofs with different interior surface emissivity (*ε*_int_). The solar irradiance is set to be 1000 W m^−2^. For both calculations, the atmospheric temperature (*T*_atm_) and room temperature (*T*_room_) are set as 35 °C and 26 °C, respectively

The international consensus is that reaching carbon emission peak and subsequent carbon neutrality are to be considered as the main global challenges in the contemporary decades. Active temperature control devices, such as air conditioners, are widely used for thermal management of enclosures including buildings and vehicles, but meanwhile, consume a considerable part of energy. In newly built buildings, the heating, ventilation, and air conditioning systems account for about 44% of total building energy consumption^1^. For electric vehicles, a 53.7% reduction in the driving range can be attributed to the air conditioning system^2^. Maintaining a suitable internal air temperature with minimal energy consumption has thereby become a long-pursued goal for the state-of-art actively temperature-regulated enclosures. Generally, the roofs of vehicles and buildings absorb 20% to 95%^3^ of the incoming sunlight, and exchange heat profoundly with the external air whose temperature can reach over 35 °C during hot seasons, which significantly increases the fuel consumption of the air conditioning system (Fig. 1a). Therefore, an advanced photonic design for the existing roofs with simultaneous thermal regulation and aesthetic functions could provide a solution for reducing the active power consumption^4–8^.

Conventional roof-based thermal management systems place a thermally insulated blanket in the interlayer of the roof to block heat transfer between the exterior and interior surfaces^9^. However, a substantial amount of heat input on the sun-facing exterior surface can still tunnel into the enclosure via conduction, convection, and radiation of the highly-emissive interior surface; besides, the room space and the aesthetics are sacrificed. Another feasible energy-saving strategy is surface passive radiative cooling. By reflecting the radiation of the sun (~6000 K) and emitting thermal radiation to the cold outer space (~3 K), the surface temperature can be reduced^10–25^. However, the sole exterior surface passive radiative cooling cannot fully utilize the double-sided roof’s capability for managing the energy exchange between the enclosure and the external space; in addition, the colors of enclosure surfaces are also required to be preserved for atheistic purposes^26–36^. For radiative cooling of a passive enclosure whose internal temperature is higher than the roof, a Janus emitter with high interior surface emissivity has also been proposed^37^. However, it is inapplicable to the commonly existing actively temperature-regulated enclosure whose temperature is lower than the roof in summer, as the roof itself transfers heat to the enclosure and adds considerable heat load to the air conditioning systems (Fig. 1b). Consequently, advanced passive cooling technology with facile integration with existing actively temperature regulated and the decorative enclosure has so far slipped out of the research at status quo.

In this work, we present a photonic-engineered thermal management strategy that incorporates an enhanced color-preserving radiative cooling (ECRC) system into the existing actively temperature-regulated and decorative enclosure (Fig. 1c). This strategy is implemented by facilely coating existing decorative roof with two photonic-engineered films having distinctive wavelength-selectivity: (1) a transparent (at visible wavelength) and mid-infrared (MIR) reflective film as the interior surface for minimizing heat load; and (2) a transparent (at visible wavelength), near-infrared (NIR) reflective, and MIR broadband emitting film as the exterior surface for reducing solar heat input and enhancing radiation power (Fig. 1d). With the ECRC thermal management strategy, the enclosure temperature can be lowered by 5.5 °C/4.0 °C/2.3 °C/5.8 °C for green/red/white/black roofs compared to the ordinary enclosure on normal summer days. Moreover, a power-saving of up to 63% for active coolers of the enclosure is demonstrated by measuring the heat flux compared to an ordinary enclosure when the set temperature is around 26 °C. This photonic-engineered dual-side thermal management strategy for facile integration with the existing actively temperature-regulated enclosures suggests avenues for enhancing the properties of nanophotonic devices toward energy conservation.

## Results

### Theoretical analysis

In this study, we consider the typical scenario of an actively temperature-regulated enclosure with a decorative roof in summer, where solar heat input cannot be totally avoided due to colors (Fig. 1a). To start with, we use a one-dimensional steady-state model to perform the theoretical analysis and an energy flow diagram to illustrate heat transfer processes (Fig. 1b). The overall system can be divided into three regions: the roof, the internal, and external space. The thermal exchange between the internal and external space is mediated by the roof with interior/exterior surfaces. In the external space, the sun (6000 K), the outer space (3 K), and the atmosphere (~ 300 K) are three fundamental thermodynamic resources, while in the internal space, the air conditioner is a common cold source. According to the second law of thermodynamics, the net heat flow in the diagram is always from the top-down, hence the vertical positions can determine the direction and the intensity of the heat flow in the system. The thermal balance for the roof can be written as

1 $$q_{{{{\mathrm{in}}}}} = q_{{{{\mathrm{solar}}}}} - q_{{{{\mathrm{out}}}}}$$

where $q_{{{{\mathrm{solar}}}}}$ is the solar power absorbed by the exterior surface, which is proportional to the solar spectral absorptivity of the exterior surface (*α*_ext_). The net outward power from the exterior surface to the atmosphere and the outer space $q_{{{{\mathrm{out}}}}}$ and the net inward power from the interior surface to the room $q_{{{{\mathrm{in}}}}}$ can be expressed as:

2 $$q_{{{{\mathrm{out}}}}} = q_{{{{\mathrm{rad}}}}}(T_{{{{\mathrm{ext}}}}},T_{{{{\mathrm{atm}}}}}) + q_{{{{\mathrm{rad}}}}}(T_{{{{\mathrm{ext}}}}},T_{{{{\mathrm{space}}}}}) + q_{{{{\mathrm{con}}}}}(T_{{{{\mathrm{ext}}}}},T_{{{{\mathrm{atm}}}}})$$

3 $$q_{{{{\mathrm{in}}}}} = q_{{{{\mathrm{rad}}}}}(T_{{{{\mathrm{int}}}}},T_{{{{\mathrm{room}}}}}) + q_{{{{\mathrm{con}}}}}(T_{{{{\mathrm{int}}}}},T_{{{{\mathrm{room}}}}})$$

Here, *q*_rad_(*T*_1_, *T*_2_) is the net radiant heat transfer between two objects. *q*_con_(*T*_1_, *T*_2_) is the conductive and convective heat transfer between two objects. *T*_ext_, *T*_int_, *T*_atm_, *T*_space_, and *T*_room_ are the temperatures of the exterior surface, the interior surface, the atmosphere, the outer space, and the room, respectively (see Supplement 1 and Fig. S1 for details).

Accordingly, the exterior surface of the roof should have two major optical properties: 1) A suitably high solar reflectance *r*_ext_. Typically, the solar irradiance power density reaches 1000 W m^−2^ under AM1.5 condition, and the proportions of ultraviolet, visible, and infrared are 8.7%, 43%, and 48.3%, respectively^38^. Considering aesthetic factors, an ideal exterior surface should have high VIS transmittance and high UV and NIR reflectance (Fig. 1d), which is usually called the “hot mirror”. We calculate the exterior surface temperature of roofs *T*_ext_ illuminated by different solar irradiances (*q*_solar_) with or without the hot mirror (Fig. 1e). The results show that the exterior surface temperature can be reduced by 21 °C under 1000 W m^−2^ solar irradiance with the hot mirror compared to that without it. 2) A high MIR emissivity *ε*_ext_. As the emission power *q*_rad_ released by an emitter can be approximately taken as *q*_rad_ = *σε*_ext_*T*^4^, a high *ε*_ext_ corresponds to high radiative cooling power (~ 520 W m^−2^ when *T* = 310 K). We also compare the broadband emitter (BE) and selective emitter (SE) for radiative cooling. The former absorbs while the latter reflects the atmospheric radiation in non-atmospheric window bands (Fig. 1d)^15,39–42^; therefore, the former has a stronger heat exchange effect with the environment (atmosphere and outer space) and can avoid overcooling^24^. The results show that the broadband emitter (both with and without a hot mirror) maintains a lower exterior surface temperature than the selective emitter, and thus the broadband emitter is chosen in the design (Fig. 1e).

As for the interior surface, a radiant barrier with a low MIR emissivity *ε*_int_ is chosen in the design to decrease the heat load from the interior surface to the room. Since the internal radiative heat transfer is cut off, the exterior surface temperature increases, which enhances the outward thermal emission *q*_rad_(*T*_ext_) and cooling effects for the whole enclosure. We calculate the net outward power from the roof to the external space *q*_out_ and the net inward power from the roof to the internal space *q*_in_ of roofs with different interior surface emissivity *ε*_int_ (Fig. 1f). The result shows that the net power to the internal space *q*_in_ decreases as emissivity decreases, while that to the external space *q*_out_ increases. Notably, compared to a conventional roof with an emissive interior surface and a solar absorptive exterior surface, a roof with an ideal ECRC can reduce the inward heat load by 81% (Fig. 1f). In short, an ideal ECRC should be engineered with dual-side visible to infrared optical properties to realize synergistically enhanced thermal management.

### Experimental design

**Fig. 2:**
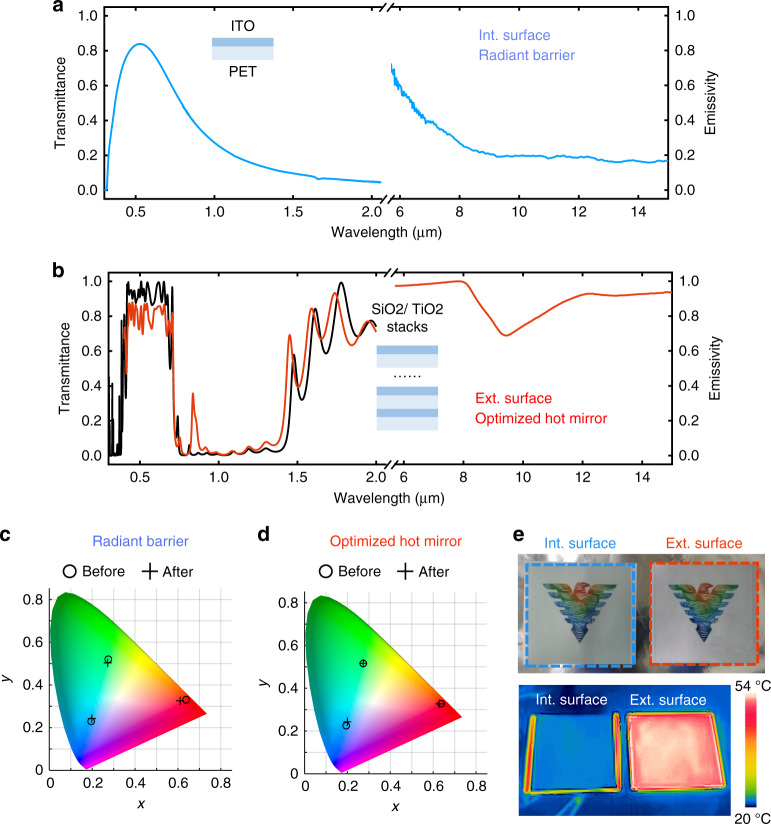
Optical properties of the interior and exterior surfaces of the ECRC. **a** Transmittance and emissivity spectra of the interior surface coating (ITO-PET film). **b** Transmittance and emissivity spectra (red) of the exterior surface coating (SiO_2_/TiO_2_ stacks film). The black line is the simulation result. **c, d** Colors in the CIE 1931xy color space map before and after (c) radiant barrier, (d) optimized hot mirror covering. **e** Optical image (upper part) and IR image (lower part) of the patterns covered by the two surfaces

Based on the analysis above, we design and fabricate the ECRC films to demonstrate the synergistically enhanced cooling for an actively temperature-regulated enclosure (see Methods for detailed fabrication). The interior surface is a thin layer of transparent indium tin oxide (ITO) on a polyester (PET) film. Due to its high carrier concentration, the ITO film exhibits excellent electrical conductivity, thus having low MIR emissivity (*ε*_6-20μm_ ~ 0.21, @ *T* = 303 K) in the mid-infrared band, while still maintaining high transmittance (*T*_0.4–0.8μm_ ~ 0.73) in the visible band (Fig. 2a). The exterior surface consists of 30 layers of titanium dioxide (TiO_2_) and silicon dioxide (SiO_2_). Careful tuning of the thickness of each layer (see Supplement 2, Fig. S2 and Fig. S3 for details) results in high visible transmittance (*T*_0.4–0.8 μm_ ~ 0.74), high NIR reflectance (*R*_0.8–2.5 μm_ ~ 0.87), as well as broadband high MIR emissivity (*ε*_6–20 μm_ ~ 0.88 @ *T* = 303 K) (Fig. 2b), which, in turn, makes its optical properties superior compared to the commercially available hot mirrors (Fig. S4).

The ECRC can be integrated with the existing decorative roofs, simply by covering the ITO film on the interior surface and the SiO_2_/TiO_2_ stack film on the exterior surface. The high transparency of the two surfaces preserves the original color of the covered objects (Fig. 2c, d, “Methods”). No obvious color distortion can be observed for the colored patterns covered by both surfaces (Fig. 2e). In the IR camera, the pattern covered by the interior surface presents a low radiation temperature, indicating suppressed heat emission; in contrast, that covered by the exterior surface shows high a radiation temperature, revealing large radiant energy.

## Experimental results

**Fig. 3:**
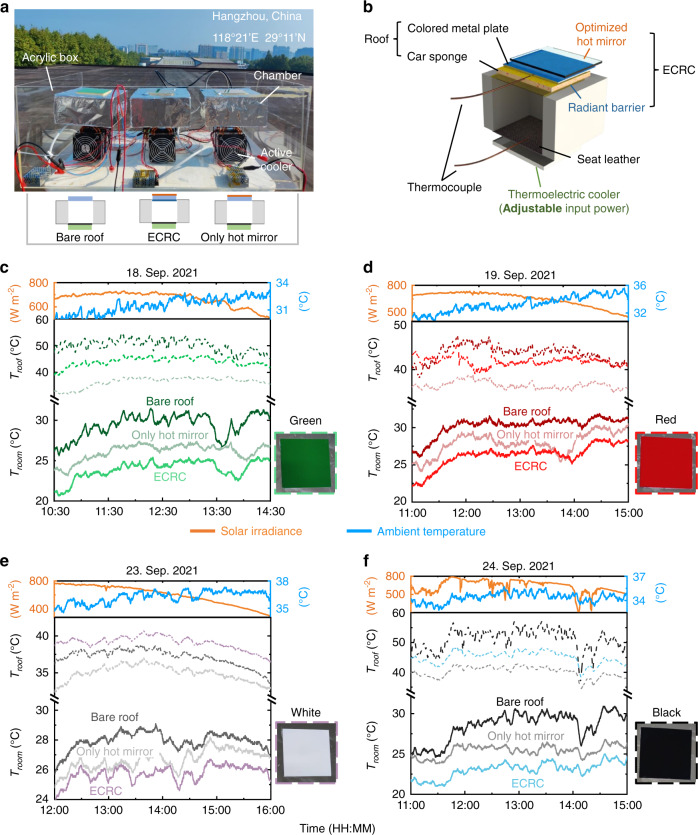
The outdoor cooling experiment of the ECRC for actively temperature regulated enclosures. **a** Photograph of the measuring setup and diagram of the three experimental groups: bare roof, roof with ECRC, and roof with only hot mirror. **b** Schematic diagram of the testing chamber. A power-adjustable thermoelectric cooler is placed at the bottom of the chamber for active temperature control. **c**–**f** Real-time room temperature *T*_room_ (solid line) and roof temperature *T*_roof_ (dashed line) in the outdoor test of the green (**c**), red (**d**), white (**e**), and black (**f**) roofs. Upper part: real-time solar irradiance (orange, left *y*-axis) and ambient temperature (blue, right *y*-axis)

The cooling performance of the ECRC for an actively temperature-regulated enclosure is experimentally characterized in an outdoor environment in Hangzhou, China. The apparatus consists of three hollow chambers equipped with the same active coolers and covered with different types of roofs: (1) bare roof, (2) roof with ECRC, and (3) roof with only hot mirror (Fig. 3a). A longitudinal section diagram of the enclosure is shown in Fig. 3b. To simulate an enclosure with active temperature control, a power-adjustable thermoelectric active cooler is placed at the bottom of the chamber. A piece of seat leather is used to mimic the internal radiation characteristics of an ordinary enclosure (Fig. 3b). The decorative roof consists of a colored metal plate and a piece of car sponge. Different colored metal plates are prepared to represent roofs with different solar absorption (Fig. S5). The sides of the three chambers are designed to be thermally insulated to study only the effect of the roof. The chambers are placed side by side in an acrylic box on a white table and face the clear sky (Fig. 3a). A pyranometer and an instrument shelter are placed aside to record solar irradiance, ambient temperature, wind speed, and air humidity. During the experiment, the temperature of the roof and room are recorded in real-time (see Methods section for further details).

To study the cooling performance of the ECRC for actively temperature regulated enclosures, we first set the same constant input power (Table S4) to the thermoelectric cooler for enclosures with the same colored roof and record the real-time room temperature *T*_room_ and roof temperature *T*_roof_ during the daytime (Fig. 3c–f). The room temperature is regulated within the range of 20 °C to 30 °C by setting different input powers for different colors, considering the difference in the absorbed solar power by the four colors as well as environmental parameters of the four days. As shown in the temperature data of Fig. 3c–f, with ECRC, the four-hour average room temperature is 5.5 °C/4.0 °C/2.3 °C/5.8 °C lower than that of the bare-roof for green/red/white/black colors, indicating a remarkable enhanced cooling performance of the ECRC for actively temperature regulated enclosures with different colored roofs. The black roof group exhibits the largest room temperature difference between roof-with-ECRC and bare-roof due to the highest absorption in the visible band. We also test the cooling performance of the roof-with-only-hot-mirror, and the results show that the room temperature is higher than that of the roof-with-ECRC for the four colors, because the extra radiant barrier under the roof for the ECRC blocks the radiation to the internal space. It is noted that for the roof-with-ECRC, the roof temperature is higher than that of the hot-mirror-only (Fig. 3c–f) due to blocking of inward radiation channel and thereby enhanced outward radiative cooling for the enclosure as a whole. Furthermore, for white color, the roof temperature of the roof-with-ECRC is even higher than that of the bare-roof (Fig. 3e). Under this circumstance, the exterior surface of the roof has minimal heat input (high *r*_solar_ of white roof), and net heat loss of the bare-roof is larger than that of the roof-with-ECRC owing to the downward radiation, which matches well with the calculated results (Fig. S6).

**Fig. 4:**
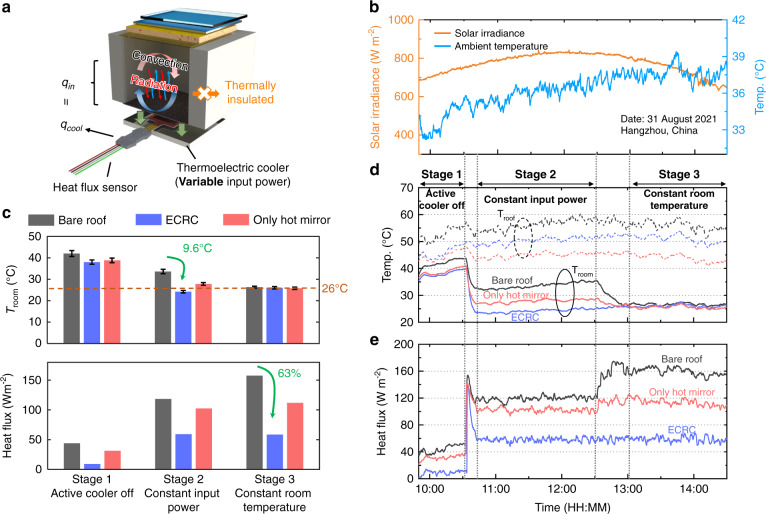
Experiment on the energy-saving effect of the ECRC for active enclosures at three stages: Active cooler off (stage 1), constant input power (stage 2), and constant room temperature (stage 3). **a** Schematic diagram of the actively temperature regulated enclosure with a heat flux sensor. The heat load of the roof to the internal space (convection plus radiation) is equal to the cooling power of the active cooler at equilibrium. **b** Real-time solar irradiance (orange) and ambient temperature (blue). **c** Room temperature (upper part) and heat flux (lower part) of the bare-roof (dark gray), roof-with-ECRC (bluish-violet), and roof-with-only-hot-mirror (pink) at three stages. Data are means ± s.d. **d** Real-time room temperature (solid line) and roof temperature (dashed line) of the three groups at three stages. The stages are separated by dash-dot lines, the positions of which are estimated by whether the heat flux is stable or not. **e** Real-time heat flux of the three groups

To study the energy-saving effect of the ECRC for the actively temperature regulated enclosures, we set variable input power (Table S4) and use a heat flux sensor to record the cooling power (*q*_cool_) of the active coolers (Fig. 4a, Method). Note that this active cooling power is not equal to the input power of the cooler due to inevitable non-cooling power consumption like ohmic loss. In thermal equilibrium, the heat load from the interior surface to the room (*q*_in_) can be considered equal to the cooling power of the active coolers as the sides of the chamber are thermally insulated (Fig. 4a). Here, we use blue color for the roof and set three groups (same as those in the first experiment). The room temperature, roof temperature, environmental parameters, and active cooling power are recorded in real-time (Fig. 4b, d). The whole experiment includes three stable stages (indicated by constant heat flux in Fig. 4e), namely ‘Active cooler off’, ‘Constant input power’, and ‘Constant room temperature’, separated by two transitional periods (Fig. 4d). At stage 1, the active coolers are off and there is only passive heat dissipation. The results show that the roof-with-ECRC has the lowest room temperature due to the minimum heat load from the roof to the room, as evidenced by the minimum heat flux (Fig. 4c). At stage 2, same constant power is added to the thermoelectric cooler for the three groups (Fig. 4d). The average room temperature of the roof-with-ECRC (24.2 °C) is 9.6 °C/3.6 °C lower than that of the bare-roof /roof-with-only-hot-mirror (33.8 °C/27.8 °C). The heat fluxes of the roof-with-ECRC, the bare-roof, and the roof-with-only-hot-mirror are 59.1 W m^−2^, 118.5 W m^−2,^ and 102.4 W m^−2^, respectively (Fig. 4c), proving that the ECRC can significantly reduce the heat load to the room and enhance the cooling performance. At stage 3, the room temperatures of the three groups are all adjusted to about 26 °C (often set for the air conditioner in summer) by adding variable input power to the thermoelectric cooler (Fig. 4d). The average heat fluxes of the roof-with-ECRC, the bare-roof, and the roof-with-only-hot-mirror are 58.4 W m^−2^, 157.7 W m^−2,^ and 111.9 W m^−2^, respectively (Fig. 4c). As a result, the ECRC can reduce the heat load, thereby achieving a power-saving of up to 63% for active coolers compared to the bare-roof when the target room temperature is about 26 °C. The prominent reduction in cooling power of the ECRC also indicates its remarkable energy-saving ability (Fig. 4e).

## Discussion

To conclude, we present a photonic-engineered dual-side thermal management strategy for reducing active power consumption of the existing temperature-regulated enclosure (including buildings, vehicles, greenhouses, etc.) without sacrificing its aesthetics. Firstly, passive radiative cooling is integrated with the existing actively temperature-regulated enclosure and a power-saving of up to 63% is demonstrated experimentally by measuring the heat flux. Second, the independent radiation control for both the interior and exterior surfaces of the enclosure provides a clear guideline for exploiting the full potential of control over the energy exchange among the enclosure, the external space, and the active coolers with photonics. Therefore, interfaces including side walls and windows that involve these two kinds of energy transfer can be applied with the ECRC strategy. Third, the proposed strategy is implemented facilely by coating the existing interior and exterior surfaces of the enclosure with two photonic-engineered films, preserving their original properties including aesthetics with only slight modification, which further extends its applicability on various decorative or even transparent surfaces. Last, the facile fabrication procedure makes the two photonic-engineered transparent films (hot mirror and radiant barrier) suitable for low-cost scalable manufacturing. We also theoretically evaluate the feasibility of the ECRC strategy for large-scale building applications, which also presents considerable cooling performance (Supplement 3 and Fig. S7). Ultimately, the innovative photonic strategy for thermal management indicates its potential for energy conservation in various scenarios requiring thermal management^42–61^, which, in turn, represents a new paradigm toward reducing carbon emission and mitigating the greenhouse effect.

## Materials and methods

### Sample fabrication and preparation

The optimized hot mirror is prepared by alternatively depositing SiO_2_ and TiO_2_ films on a quartz glass substrate with an electron beam evaporation system, and the deposition rates are 0.3 nm s^−1^ for TiO_2_ and 0.5 nm s^−1^ for SiO_2_. The radiant barrier is a 200-μm-thick ITO-PET film bought from Southern China Xiang’s Science & Technology, and is fabricated through magnetron sputtering, followed by thermal annealing (a temperature range of 80–120 °C). The square resistance of the ITO film is ~6 Ω.

### Optical measurements

Solar (UV, VIS, and NIR) transmission and reflection spectra are measured using a universal measurement spectrophotometer (Agilent, Cary7000) equipped with an integrating sphere. The incident angle of the light source onto the samples is about 8°. MIR emissivity is measured with a Fourier transform infrared spectrometer (Bruker, Vertex 70) with a room-temperature doped triglycine sulfate (DTGS) detector. Black soot deposited on a gold-coated silicon wafer using a burning candle is used as a reference. All samples are fixed on the same heater connected with a temperature controller. The temperature for measurement is set at 70 °C.

### Color preparation and characterization

The colored metal plates are prepared by spray painting on 0.5-mm-thick aluminum plates using aerosol paints (J2A, SANO). Colors of all objects are measured using a color matching tool (ColorReader, datacolor) calibrated with a white tile.

### Simulated active enclosure construction

The simulated active enclosure consists of the roof, the hollow chamber, and the active cooler (Fig. 3b). The roof includes a colored metal plate and a piece of car sponge. The car sponge is a piece of 2-mm-thick sponge (Polyester, PET) attached with a layer of 0.1-mm-thick flannel (Polyester), and the flannel is facing the internal space during the experiment. The chamber is a 20 cm × 20 cm × 10 cm foam box (expanded polystyrene, EPS) hollowed out in the middle and the size of the hollow part is 10 cm × 10 cm × 10 cm. The external and internal sides of the chamber are adhered with aluminum foils. The bottom of the chamber is a piece of 0.6-mm-thick seat leather (Polyurethane, PU) to mimic the internal radiation characteristics of an ordinary enclosure. The active cooler is a Peltier semiconductor cooling plate connected to a fan and driven by a DC power supply. The sizes of the metal plate, the car sponge, the seat leather, and the thermoelectric cooler are all 10 cm × 10 cm.

### Thermal measurement

The temperature measurements are performed with thermocouples (Omega, SA-1K), and the heat flux measurements are performed with a heat flux sensor (FHF02, Hukseflux). A recorder (KSB24A0R) is used to acquire the real-time temperature and heat flux. For measuring the roof temperature, the thermocouples are placed between the metal plate and the car sponge; and for measuring the room temperature, the thermocouples are attached to the upper surface of the seat leather. The heat flux sensor is placed between the seat leather and the thermoelectric cooler. During the thermal measurement, three simulated active enclosures are placed facing the clear sky without any tilt and exposed to wind flow. A pyranometer and an instrument shelter are used to record real-time solar irradiance power density, ambient temperature, relative humidity, and wind speed, respectively.

## Supplementary information

Supplementary Information for Color-preserving passive radiative cooling for an actively temperature regulated enclosure
