# Supplementary material for: Color-preserving passive radiative cooling for an actively temperature-regulated enclosure
Source: Light Sci Appl. 2022 May 4;11:122. doi: 10.1038/s41377-022-00810-y (PMC9068694; doi:10.1038/s41377-022-00810-y)
Supplement: Supplementary file 1 — Supplementary Information for Color-preserving passive radiative cooling for an actively temperature regulated enclosure [file 41377_2022_810_MOESM1_ESM.docx]

Supplementary Information for

**Color-preserving passive radiative cooling for an actively temperature regulated enclosure**

*Yining Zhu^1^, Hao Luo^1^, Chenying Yang^1^, Bing Qin^1^, Pintu Ghosh^1^, Sandeep Kaur^1^, Weidong Shen^1^, Min Qiu^2,3,*^, Pavel Belov^4^, and Qiang Li^1,*^*

^1^State Key Laboratory of Modern Optical Instrumentation, College of Optical Science and Engineering, Zhejiang University, Hangzhou 310027, China

^2^Key Laboratory of 3D Micro/Nano Fabrication and Characterization of Zhejiang Province, School of Engineering, Westlake University, 18 Shilongshan Road, Hangzhou 310024, Zhejiang Province, China

^3^Institute of Advanced Technology, Westlake Institute for Advanced Study, 18 Shilongshan Road, Hangzhou 310024, Zhejiang Province, China

^4^Department of Physics and Engineering, ITMO University, Russia

^*^E-mail: qiangli@zju.edu.cn; qiu_lab@westlake.edu.cn

Supplement 1. Heat transfer model analysis

A one-dimensional steady state heat transfer model is used to determine the energy flow among the roof, the internal space, and the external space of an active enclosure. In this model, solar illumination, thermal radiation, conduction, and convection are included to simulate the cooling power consumption of the active coolers (Fig. S1).

The energy balance at the exterior surface of the roof can be written as

 (S1)

Energy balance at the interior surface of the roof can be given by

 (S2)

The temperature of the exterior and the interior surface of the roof then can be calculated by combining the above two equations.

The heat load from the roof interior to the room can be termed as

 (S3)

The cooling power from the active cooler is equal to the heat load in thermal equilibrium, which can be expressed as

 (S4)

Here, $q_{solar}$ is the solar power absorbed by the exterior surface, $q_{\text{atm}}$ is the absorbed radiation heat flux from the atmosphere, $q_{\text{rad\_out}}$ is the radiation heat flux from the exterior surface of the roof, $q_{\text{conv\_out}}$ is the convective heat flux from the exterior surface of the roof to the atmosphere, $q_{\text{cond}}$ is the conductive heat flux from the interior surface to the exterior surface of the roof, $q_{\text{rad\_in}}$ is the radiation heat flux from the interior surface of the roof, $q_{\text{rad\_room}}$ is the radiation heat flux from the room, $q_{\text{conv\_in}}$ is the convective heat flux from the interior surface of the roof to the room. All parameters are calculated with the equations (S4-S11) and Table S1

Expressions of terms involved in heat transfer model analysis

$q_{\text{solar}}=\int\text{d}\lambda\cdot I_{\text{AM1.5}}(\lambda)\cdot\alpha_{\text{ext}}(\lambda)$ (S4)

$q_{\text{atm}}=\int_{0}^{\frac{\pi}{2}} \text{d}\theta\cdot\pi\sin(2\theta)\int\text{d}\lambda\cdot I_{\text{BB}}(T_{\text{atm}},\lambda)\varepsilon_{\text{ext}}(\lambda)(1-\tau_{\text{MIR}}(\lambda)^{1/\cos(\theta)})$ (S5)

$q_{\text{rad\_out}}=\varepsilon_{\text{ext}}\sigma T_{\text{ext}}^{4}$ (S6)

$q_{\text{conv\_out}}=h_{\text{ext}}\cdot(T_{\text{ext}}-T_{\text{atm}})$ (S7)

$q_{\text{cond}}=k_{\text{r}}\cdot(\frac{T_{\text{ext}}-T_{\text{int}}}{t_{\text{r}}})$ (S8)

$q_{\text{rad\_in}}=\varepsilon_{\text{in}}\sigma T_{\text{in}}^{4}$ (S9)

$q_{\text{rad\_room}}=\sigma T_{\text{room}}^{4}$ (S10)

$q_{\text{conv\_in}}\text{=}h_{\text{int}}\cdot(T_{\text{int}}-T_{\text{room}})$ (S11)

Table S1 | Parameters used in the analysis of heat transfer model

| Symbol | Definition | Value | Unit |
| --- | --- | --- | --- |
| *I* | Spectral radiance | *I*_AM1.5_, AM1.5 solar spectrum  *I*_BB_, blackbody thermal radiation | [W m^-2^ nm^-1^] |
| *T* | Temperature | Room, *T*_room_ = 299.15  Atmosphere, *T*_atm_ = 308.15  Roof exterior surface, *T*_ext_  Roof interior surface, *T*_int_ | [K] |
| *h* | Natural convective heat transfer coefficient | Exterior surface, *h*_ext_  Interior surface, *h*_int_ | [W m^-2^ K^-1^] |
| *σ* | Stefan-Boltzmann constant | 5.67×10^-8^ | [W m^-2^ K^-4^] |
| *k* | Thermal conductivity | Roof, *k*_r_ = 0.06 | [W m^-1^ K^-1^] |
| *t* | Thickness | Roof, *t*_r_ = 2 | [mm] |
| *τ*_MIR_ | IR transmittance | Atmosphere, Spectral selective ^12^ | unitless |
| *ε* | IR emissivity | Roof exterior surface, *ε*_ext_  (with hot mirror, *ε*_ext_ = 0.88; without hot mirror, *ε*_ext_ = 0.9)  Roof interior surface, *ε*_int_  (with radiant barrier , *ε*_int_ = 0.21; without radiant barrier, *ε*_int_ = 0.92) | Unitless |
| *α* | Solar absorptivity | Roof exterior surface, *α*_ext_ | unitless |

*The convective heat transfer coefficient is derived from the experiments.


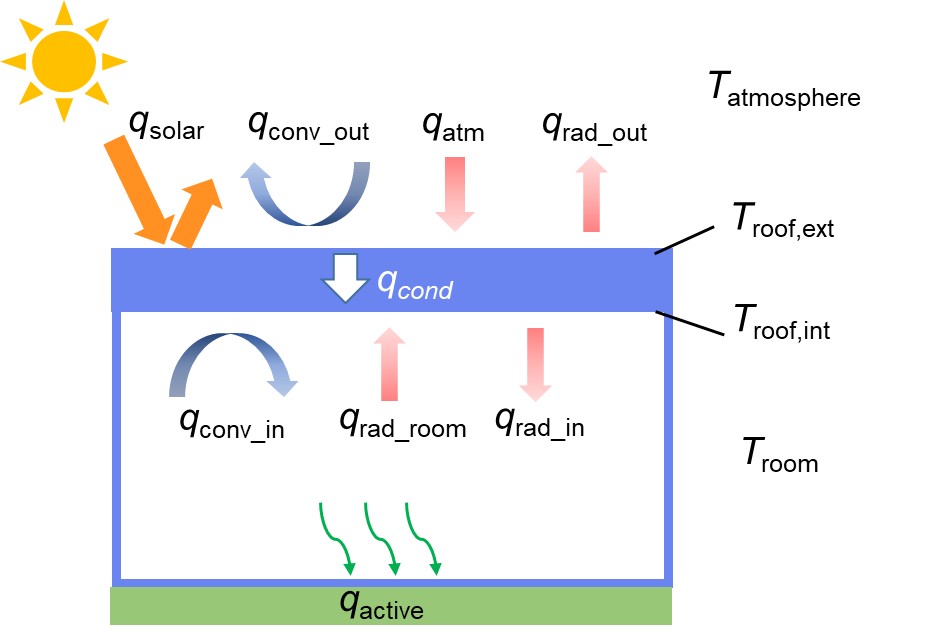


**Fig. S1 |** Schematic of a one-dimensional steady state heat transfer model of active enclosures.

Supplement 2. Optimization of the SiO_2_/TiO­­_2_ multilayers

The film design objective is to simultaneously maximize the visible transmittance and the near-infrared reflectance. To start with, we generate a periodic structure in which two materials with different refractive index are arranged alternatively (Fig. S2a). Here, we choose TiO_2_ (*n* ~ 2.3) and SiO_2_ (*n* ~ 1.4), which are commonly used for their difference on refractive index in the visible and near-infrared band (Fig. S2b). The thicknesses are adjusted to match the band equation of one-dimensional photonic (1D PhC) crystal:

$\omega_{\text{c}}\text{=}\frac{\pi c}{n_{1}d_{1}+n_{2}d_{2}}$ (S12)

where *ω*_c_ is the central frequency of the band, *d_1_* and *d_2_* are the thickness, *n*_1_ and *n*_2_ are the refractive index. According to the objective, we obtain two groups of thickness: 1) *d*_1_ = 100 nm, *d*_2_ = 180 nm, and 2) *d*_1_ = 120 nm, *d*_2_ = 220 nm, corresponding to the central wavelength (*λ*_c_) of 1000 nm and 1200 nm respectively, as shown in Fig. S2c. Then, we combine the two thickness groups and use genetic algorithm to further optimize the spectrum of the multilayers (Fig. S2c).

The layer numbers will influence both the visible transmittance and the NIR reflectance. Higher visible transmittance and higher NIR reflectance can be achieved by using more layers. The reflectance spectra for 10 layers, 20 layers, and 30 layers are shown in Fig. S3a, and calculated visible transmittance and near-infrared reflectance are shown in Fig. S3b.


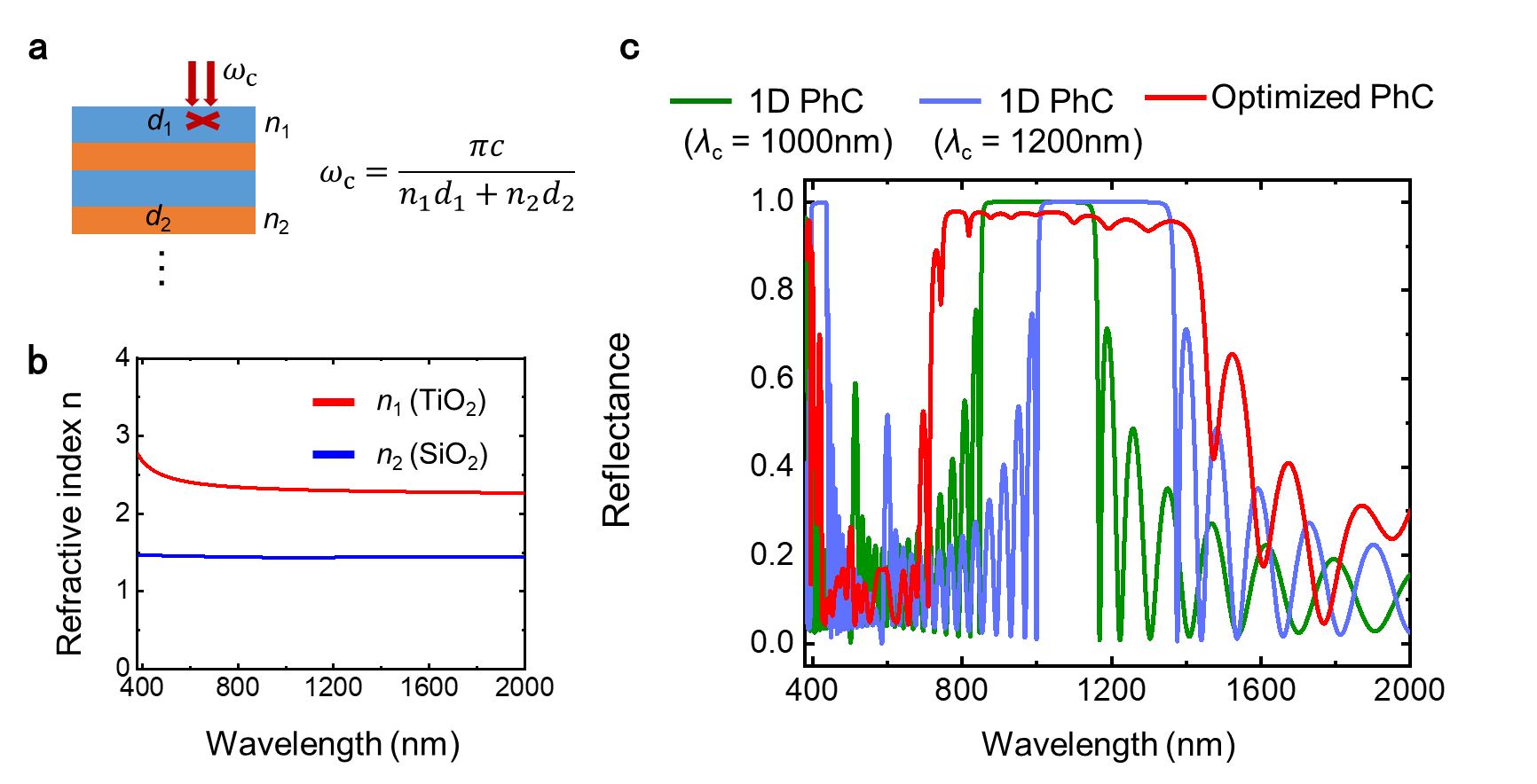


**Fig. S2 | (a)** The schematic diagram of one-dimensional photonic crystal. **(b)** Refractive index of TiO_2_ (red line) and SiO_2_ (blue line). **(c)** Reflectance spectra of the 1D PhC with central wavelength of 1000 nm (green line), 1D PhC with central wavelength of 1200 nm (blue line) and 1D PhC after optimized (red line).


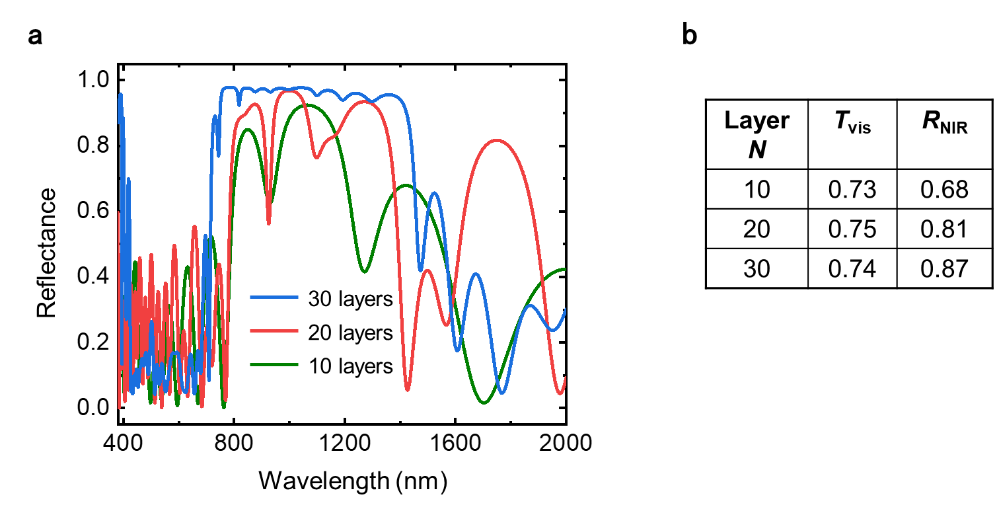


**Fig. S3 | (a)** Reflectance spectra for 10 layers, 20 layers, and 30 layers of TiO_2_/SiO_2_. **(b)** Calculated visible transmittance and near-infrared reflectance for different layer numbers.


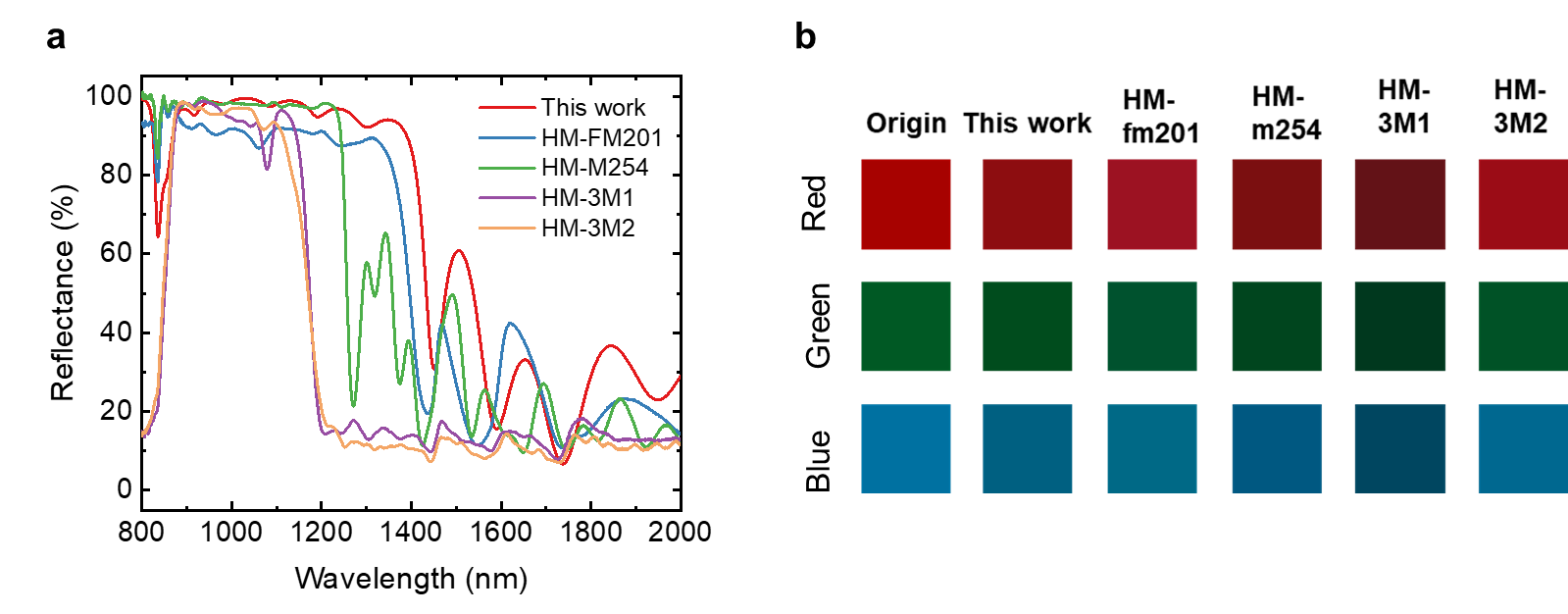


**Fig. S4 |** **Comparison with commercial hot mirrors. (a)** NIR reflectance of different hot mirrors (red: this work; blue: hot mirror, FM201, Thorlabs; green: hot mirror, M254, Thorlabs; purple: heat-insulating film, 3M ID XI003906611; orange: heat-insulating film, 3M ID XI003906561). (**b)** Colors before and after covering different hot mirrors. The color differences (Δ*E*_76_) are given in Table S2.

**Table S2 | Color difference (ΔE_76_) before and after covering different hot mirrors.**

| Δ*E*_76_ | This work | HM-FM201 | HM-M254 | HM-3M1 | HM-3M2 |
| --- | --- | --- | --- | --- | --- |
| Red | 15.5 | 19.5 | 24.4 | 39.6 | 13.5 |
| Green | 8.5 | 13.3 | 13.5 | 26.4 | 8.4 |
| Blue | 10.8 | 11.9 | 11.3 | 22 | 5.9 |


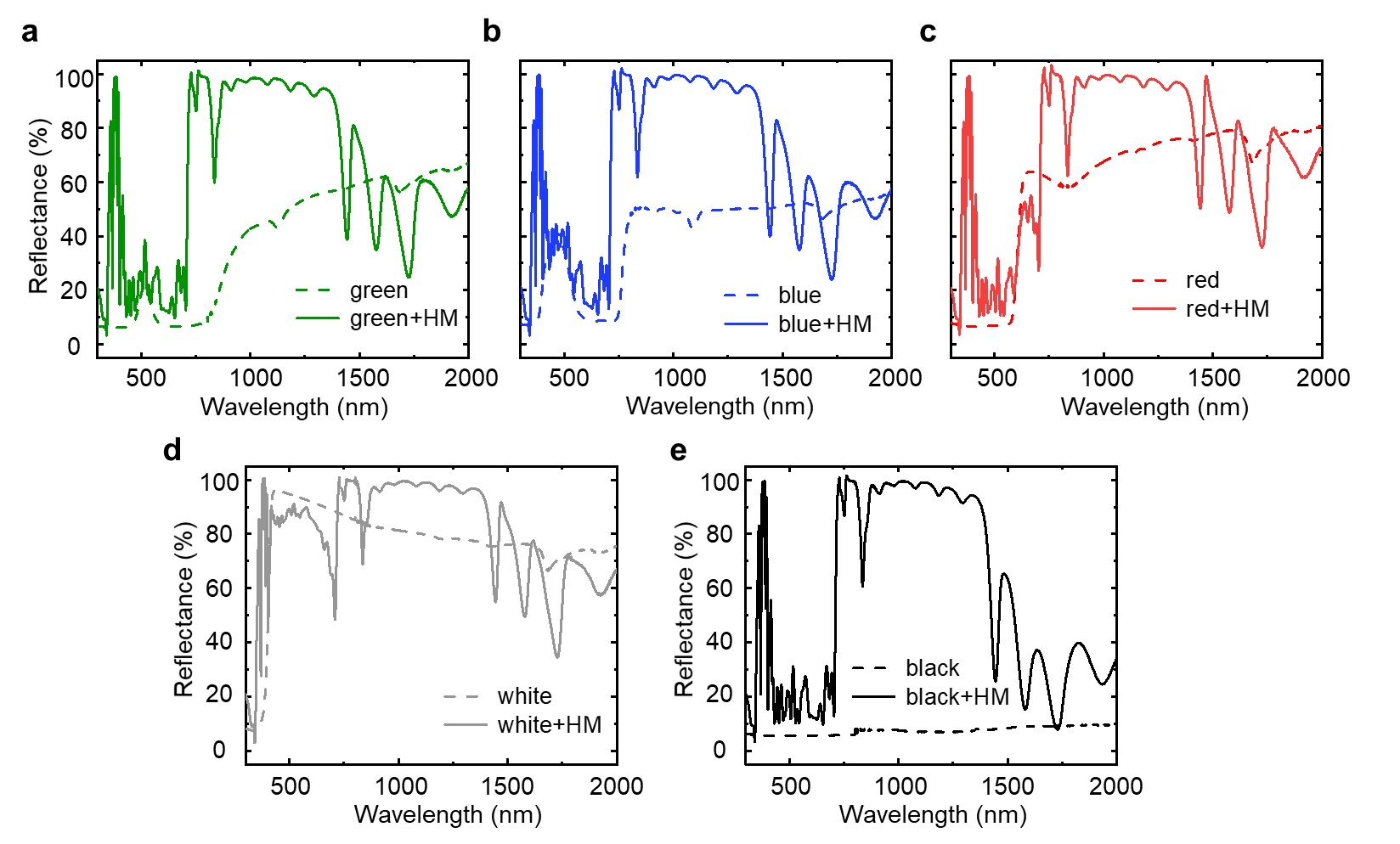


**Fig. S5** **|** Solar reflectance spectra of (**a)** green, (**b)** blue, (**c)** red, (**d)** white, and (**e)** black colored roofs before and after covering the optimized hot mirror (HM). The solar absorptivity (*α*_solar_) are given in Table S3.

**Table S3 | Solar absorptivity of different colored roofs with or without the optimized hot mirror.**

| *α*_solar_ | Green | Blue | Red | White | Black |
| --- | --- | --- | --- | --- | --- |
| w/o HM | 0.78 | 0.68 | 0.55 | 0.17 | 0.93 |
| w/t HM | 0.45 | 0.41 | 0.39 | 0.14 | 0.47 |

**Table S4 | Input power for active coolers during the experiment of different colored roofs.**

| Color/Date | Green / 9-18 | Red / 9-19 | Blue / 8-31 |
| --- | --- | --- | --- |
| Input power (W m^-2^) | 160 | 120 | Stage 2: 330  Stage 3: 1485/330/590 for  Bare/ECRC/Only hot mirror |
| Color/Date | White / 9-23 | Black / 9-24 |  |
| Input power (W m^-2^) | 110 | 195 |  |


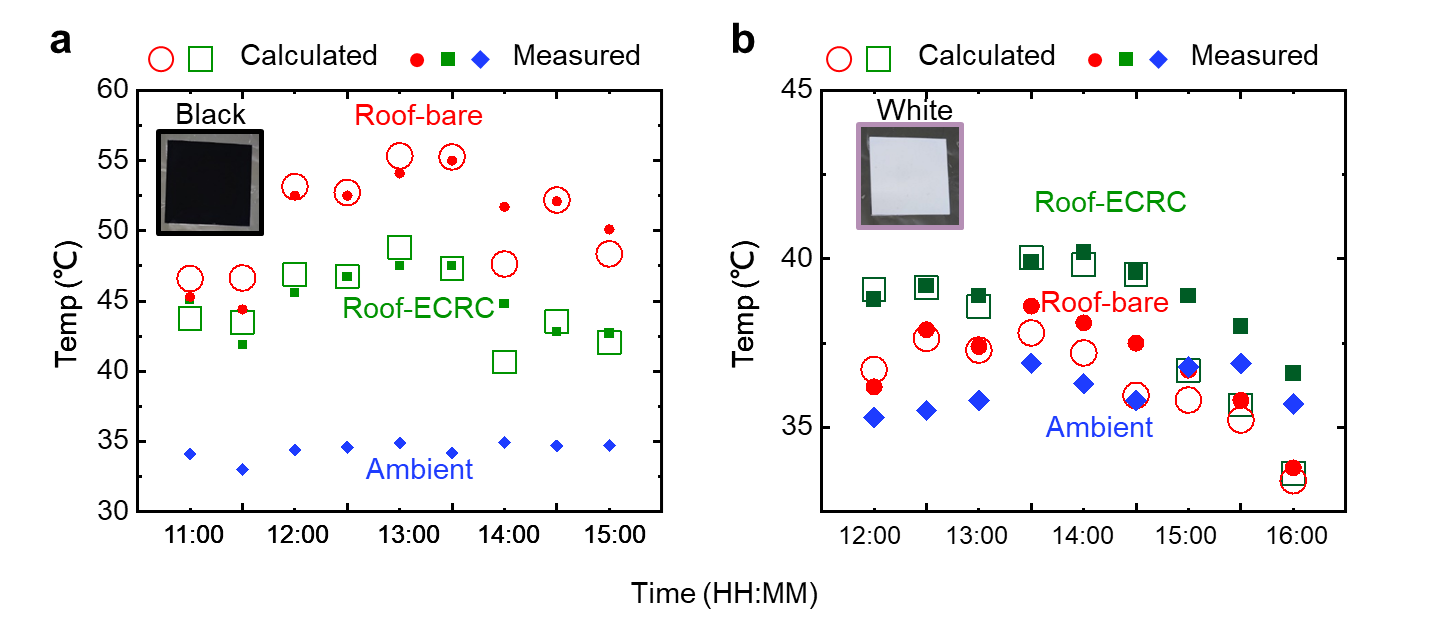


**Fig S6 |** Measured (filled dot) and calculated (hollow dot) roof temperature for the bare-roof (red circle) and the roof-with-ECRC (green square) of (**a)** black, and (**b)** white colored roofs. The blue filled dots denote the measured ambient temperature. The calculations are based on the one-dimensional steady state heat transfer model in Supplement 1.

Supplement 3. Evaluation of feasibility for large-scale building applications

To further discuss the feasibility of ECRC strategy for large-scale building applications, a two-dimensional transient heat transfer model is generated (Fig. S7a) and a commercial software (COMSOL Multiphysics 5.5) is used to calculate the results. Here the scale is defined as the side length of the simulated square enclosure. Full consideration of the natural convection inside the enclosure due to laminar flow, the heat conduction among the solid region, and the radiation exchange between the external environment and the roof exterior as well as surface-to-surface radiation inside the enclosure are included. The input solar absorptivity and infrared emissivity for exterior/interior surfaces of bare-roof and roof-with-ECRC are from the experimental results, which are shown in Fig. S7b. As shown in Fig. S7c, the cooling temperature (temperature differences between bare-roof and roof-with-ECRC) of enclosures with scales from 0.1 m to 10 m. As scale increases from 0.1 m to 2 m, the cooling temperature of the room increases about 2 ℃, while the cooling temperature of the roof has a drop less than 0.5 ℃. The results suggest that the ECRC strategy can be used for large-scale buildings as well. We also fit the time constants of the heat transfer processes for different scales as shown in Fig. S7d. The results show that the thermal diffusion inside the enclosure slows down as the scale increases, however, the speed is still faster than the change of the outdoor environment during the day.


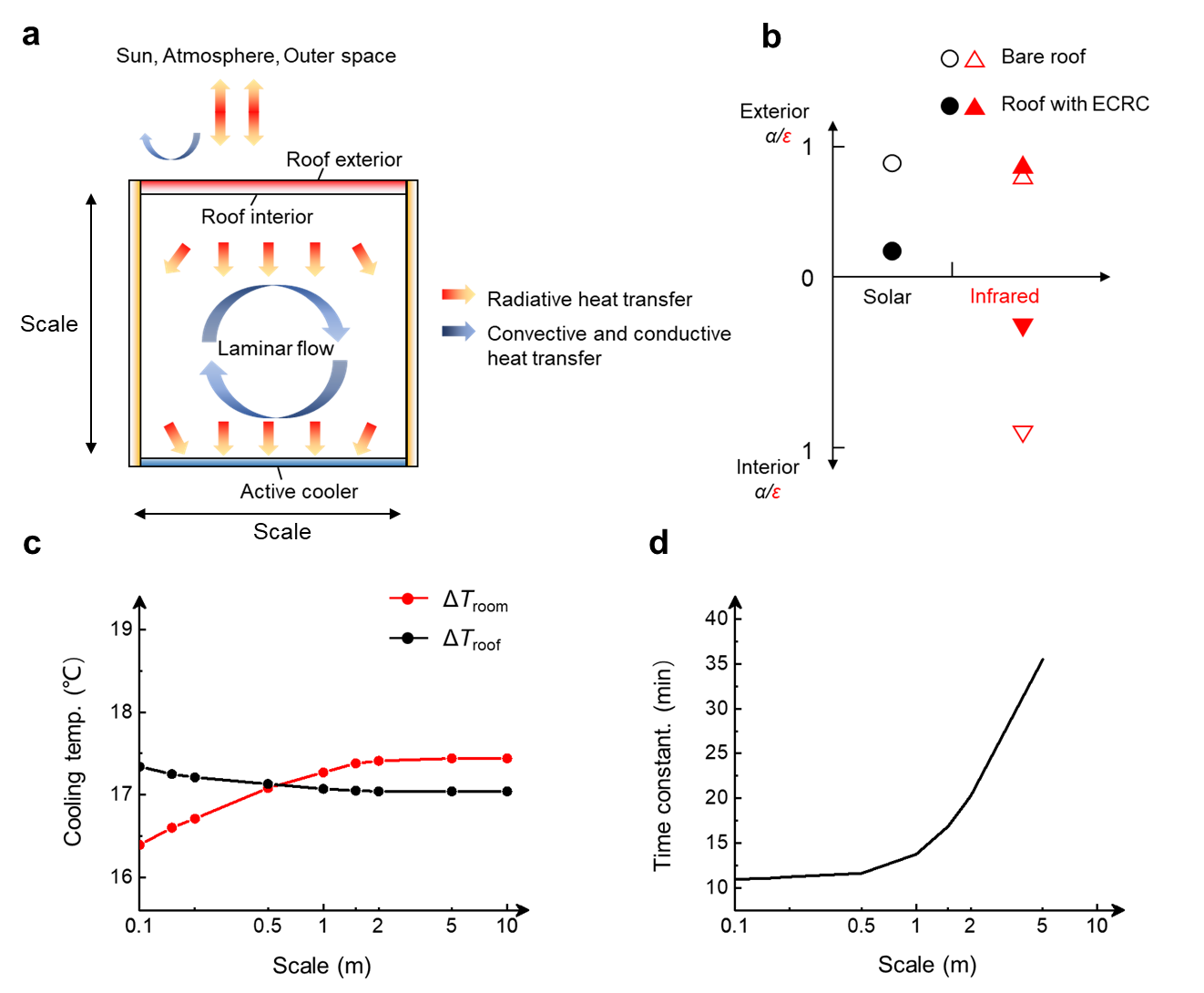


**Fig. S7 (a)** Schematic illustration of the two-dimensional model for theoretical analysis on heat transfer in enclosure. **(b)** The input solar absorptivity and infrared emissivity for exterior/interior surfaces of bare-roof and roof-with-ECRC. **(c)** Cooling temperature (temperature differences between bare-roof and roof-with-ECRC) of enclosures with scales from 0.1 m to 10 m. The red line denotes the temperature difference inside the room while the black line denotes the temperature difference at the roof. **(d)** Time constants of the heat transfer processes for different scales.

**Table S5 | The thickness of each layer of the SiO_2_/TiO_2_ stack film**

| Layer | Material | Thickness (nm) | Layer | Material | Thickness (nm) |
| --- | --- | --- | --- | --- | --- |
| 1 | TiO_2_ | 14 | 16 | SiO_2_ | 161 |
| 2 | SiO_2_ | 40 | 17 | TiO_2_ | 104 |
| 3 | TiO_2_ | 114 | 18 | SiO_2_ | 331 |
| 4 | SiO_2_ | 165 | 19 | TiO_2_ | 101 |
| 5 | TiO_2_ | 89 | 20 | SiO_2_ | 166 |
| 6 | SiO_2_ | 152 | 21 | TiO_2_ | 94 |
| 7 | TiO_2_ | 98 | 22 | SiO_2_ | 352 |
| 8 | SiO_2_ | 172 | 23 | TiO_2_ | 105 |
| 9 | TiO_2_ | 114 | 24 | SiO_2_ | 174 |
| 10 | SiO_2_ | 181 | 25 | TiO_2_ | 211 |
| 11 | TiO_2_ | 106 | 26 | SiO_2_ | 164 |
| 12 | SiO_2_ | 166 | 27 | TiO_2_ | 105 |
| 13 | TiO_2_ | 100 | 28 | SiO_2_ | 325 |
| 14 | SiO_2_ | 172 | 29 | TiO_2_ | 99 |
| 15 | TiO_2_ | 100 | 30 | SiO_2_ | 67 |
